# A new species and range extensions for three other species of pebblesnails (Lithoglyphidae, *Fluminicola*) from the upper Klamath basin, California–Oregon

**DOI:** 10.3897/zookeys.812.29205

**Published:** 2019-01-03

**Authors:** Hsiu-Ping Liu, Robert Hershler

**Affiliations:** 1 Department of Biology, Metropolitan State University of Denver, Denver, CO 80217, USA Metropolitan State University of Denver Denver United States of America; 2 Department of Invertebrate Zoology, Smithsonian Institution, Washington, D.C. 20013-7012, USA Smithsonian Institution Washington United States of America

**Keywords:** Caenogastropoda, freshwater, mitochondrial DNA, morphology, Pacific Northwest, systematics, Truncatelloidea

## Abstract

This is the fifth in a recent series of papers on the poorly known western North American pebblesnail genus *Fluminicola* (Caenogastropoda, Lithoglyphidae). Herein we clarify the taxonomic status of the currently undescribed pebblesnail fauna in the upper Klamath River drainage (UKL) based on morphologic evidence, and mitochondrial DNA sequence data from 58 UKL collection localities. We describe one new species (*F.klamathensis*) from eight UKL localities which is differentiated by mtDNA sequences and unique penial morphology, and document range extensions to the UKL for three species from closely proximal drainages (*F.fresti*, *F.modoci*, *F.multifarius*). *Fluminicolafresti* was found at a single locality along the western edge of upper Klamath Lake. *Fluminicolamodoci* and *F.multifarius* are widely distributed in the UKL; both species exhibit marked morphologic variation yet are relatively little differentiated genetically in this basin.

## Introduction

This is the fifth in a recent series of papers on the freshwater pebblesnails (Lithoglyphidae: *Fluminicola*) of the Pacific Northwest, USA. The previous contributions in this series, which treated the faunas in the Rogue–Umpqua (Hershler et al. 2017), upper Sacramento River (Hershler et al. 2007), and Snake River watersheds (Hershler and Liu 2012; Liu et al. 2013), increased the number of *Fluminicola* species from 9 to 27 and also documented large range extensions for *F.coloradoense* Morrison, 1940 and *F.multifarius* Hershler, Liu, Frest & Johannes, 2007.

The pebblesnails in the upper Klamath River drainage (UKL), California–Oregon, have been little studied historically and are currently unassigned to species (Hershler and Frest 1996). In various contract reports documenting their extensive field surveys of UKL freshwater mollusks, Frest and Johannes (1998, 2000, 2004, 2005, and other references cited therein) recognized 24 purportedly undescribed, narrowly ranging pebblesnail species from this watershed (based largely on shells and body pigmentation) and gave them provisional scientific (e.g., “*Fluminicola* n. sp. 1”) and colloquial names. Although the UKL pebblesnails have subsequently become a focus of conservation attention–e.g, four of the putative novelties recognized by Frest and Johannes were incorporated into the Northwest Forest Plan as “survey and management species” (USDA and USDI 1994; also see Frest and Johannes 1999) and three of these were petitioned (unsuccessfully) for addition to the Federal List of Threatened and Endangered Species (Curry et al. 2008; USFWS 2012)–there have been no recently published studies of this fauna aside from a molecular phylogenetic analysis which delineated a close relationship between specimens from the Link River (the outlet of Upper Klamath lake) and *F.modoci* Hannibal, 1912 from the Goose Lake basin (Hershler et al. 2007). Herein we utilize both molecular (mitochondrial DNA sequences) and morphological data to delimit the UKL pebblesnail species. The former has proved very useful in previous taxonomic studies of pebblesnails, enabling delineation of both morphologically cryptic, and morphologically variable species (Hershler et al. 2007, 2017; Liu et al. 2013).

## Methods

For this project we sequenced specimens from 58 UKL localities that were sampled in August 2012 and May and September 2013. Collections were made at three localities (Harriman Springs, Wood River south spring source, spring brook below Schoolhouse Meadow) on more than one occasion in an effort to increase sample sizes. Specimens were collected by hand or with a small sieve and preserved in 90% (non-denatured) ethanol in the field. Portions of several samples were relaxed with menthol crystals, fixed in dilute formalin, and preserved in 70% ethanol for anatomical study. Vouchers were deposited in the Smithsonian Institution’s National Museum of Natural History (USNM) collection.

Some of the collections contained multiple shell morphotypes which were sorted and analyzed separately, yielding a total of 80 samples (UKL12–UKL91). Cytochrome c oxidase subunit I (COI) and cytochrome B (cytB) sequences were obtained from 283 and 259 UKL specimens, respectively. Genomic DNA was extracted from entire snails using a CTAB protocol (Bucklin 1992); each specimen was analyzed for mtDNA individually. LCO1490 and HCO2198 (Folmer et al. 1994) were used to amplify a 709 base pair (bp) fragment of COI; cytB427F (5’TGA GGK GCN ACT GTT ATT ACT AA3’) and cytB1049R (5’GTG AAA ACT TGS CCR ATT TGC TC3’) were used to amplify a 644 bp fragment of the cytB gene. The cytB427F and cytB1049R primers were designed based on conserved regions of cytB in an alignment using previously published sequences from *Oncomelaniahupensis* (Gredler) (NC13073) and *Potamopyrgusantipodarum* (Gray) (GQ996433). Amplification conditions and sequencing of amplified polymerase chain reaction product methods were those of Liu et al. (2013). Sequences were determined for both strands and then edited and aligned using Sequencher™ version 5.4.1 (Gene Codes Corporation, Ann Arbor, MI). In order to generate easily readable topologies, one example of each unique UKL haplotype was used in the phylogenetic analyses, which were performed separately for the COI and cytB datasets. The analyses of the COI dataset also included the previously published UKL haplotypes (from a single collection locality), and sequences from 14 regional *Fluminicola* species and representatives of two other North American lithogyphid genera (*Somatogyrus*, *Taylorconcha*). Trees were rooted with *Pristinicolahemphilli* (Pilsbry) (Hydrobiidae). The cytB dataset also included sequences from 13 *Fluminicola* species (a cytB sequence is not available for *F.gustafsoni* Hershler & Liu). Given that cytB sequences are not available for other North American lithoglyphid genera, basally positioned *F.virens* was used to root the trees based on this dataset (Hershler et al. 2007; Hershler and Liu 2012). Sample codes, locality and voucher details, and GenBank accession numbers for the sequences used in the molecular phylogenetic analyses are in Suppl. material 1.

Genetic distances were calculated using MEGA7 (Kumar et al. 2016), with standard errors estimated by 1,000 bootstrap replications with pairwise deletion of missing data. MrModeltest v. 2.3 (Nylander 2004) selected the GTR + I + G model parameters as the best fit for both the COI and cytB datasets (using the Akaike Information Criterion). Phylogenetic analyses were performed using maximum parsimony (MP), maximum likelihood (ML), and Bayesian inference (BI) methods; trees were also generated using a distance method. The MP and ML analyses were performed using PAUP* v. 4.0b10 (Swofford 2003) and the BI analyses were conducted using MrBayes v. 3.2.6 (Ronquist et al. 2012). The MP analyses were conducted with equal weighting, using the heuristic search option with tree bisection reconnection branch-swapping and 100 random additions. Nodal support was evaluated by 10,000 bootstrap replicates. The ML analyses were performed using the GTR + I + G model. The optimized parameter values for COI were base frequencies of A = 0.3089, T = 0.3856, C = 0.1684, G = 0.1371; shape of gamma distribution = 1.1801; proportion of invariant sites = 0.5691. The optimized parameter values for cytB were base frequencies of A = 0.3146, T = 0.3671, C = 0.1917, G = 0.1268; shape of gamma distribution = 0.7706; proportion of invariant sites = 0.3818. A GTR distance-based neighbor-joining (NJ) tree was used as the initial topology for branch-swapping. Nodal support was evaluated by 1,000 bootstrap pseudo-replicates. For the BI analyses Metropolis-coupled Markov chain Monte Carlo simulations were run with four chains (using the model selected by MrModeltest) for 5,000,000 generations. Markov chains were sampled at intervals of 100 generations to obtain 50,000 sample points. We used the default settings for the priors on topologies and the GTR + I + G model parameters. At the end of the analyses, the average standard deviations of split frequencies were 0.005763 (COI dataset) and 0.004997 (cytB dataset) and the potential scale reduction factor (PSRF) was 1, indicating that the runs had reached convergence. The sampled trees with branch lengths were used to generate 50% majority rule consensus trees, with the first 25% of the samples removed to ensure that the chain sampled a stationary portion. For the distance analyses, HKY distances were used to generate neighbor-joining (NJ) trees (Saitou and Nei 1987).

We also studied pertinent specimens in the USNM collection, including UKL material collected by Frest and Johannes that was acquired during the planning stage of this project. The total number of shell whorls (WH) was counted for each specimen; and the height and width of the entire shell (SH, SW), body whorl (HBW, WBW), and aperture (AH, AW) were measured from camera lucida outline drawings (Hershler 1989). Photographs of alcohol preserved specimens (that had been anaesthetized with menthol crystals prior to fixation) were taken using a Coolpix 990 mounted on an Olympus SZX12 dissecting microscope. Other methods of morphological study were routine (Hershler et al. 2007). Shell descriptive statistics were generated using Systat for Windows 11.01 (SSI 2004).

## Results

**Figure 1.**
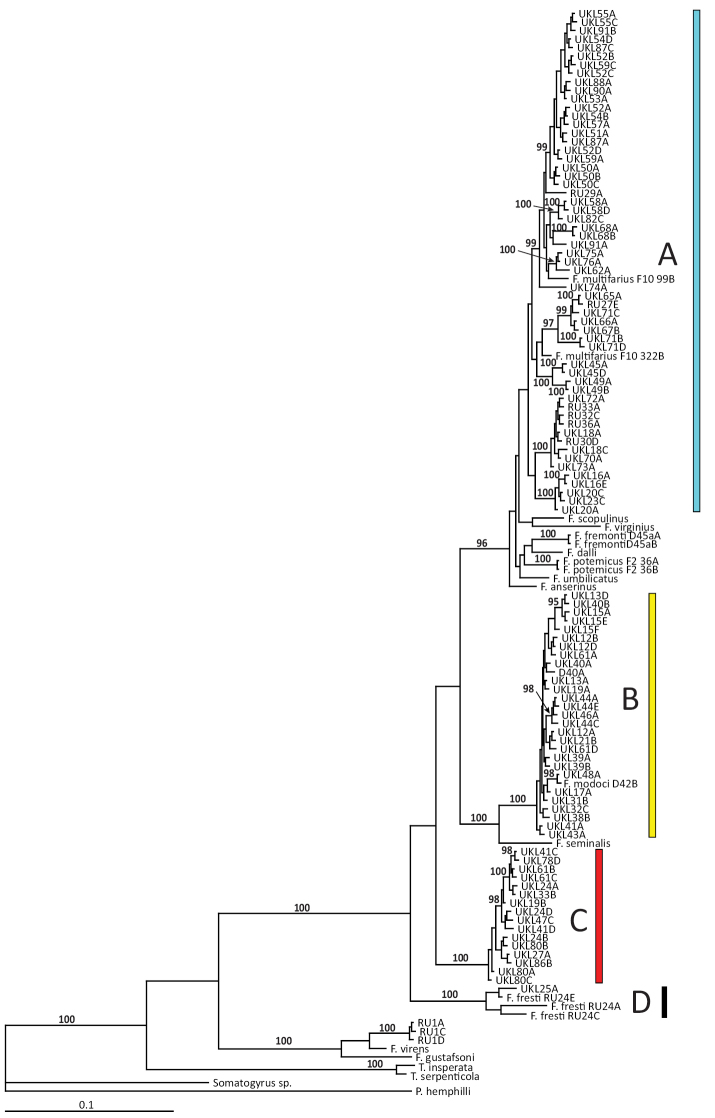
Bayesian tree based on the COI dataset. The four clades (**A–D**) containing UKL haplotypes are color coded as in Figure 2. Posterior probabilities for nodes are shown when ≥ 95%. Specimen codes are from Suppl. materials 1–3.

**Figure 2.**
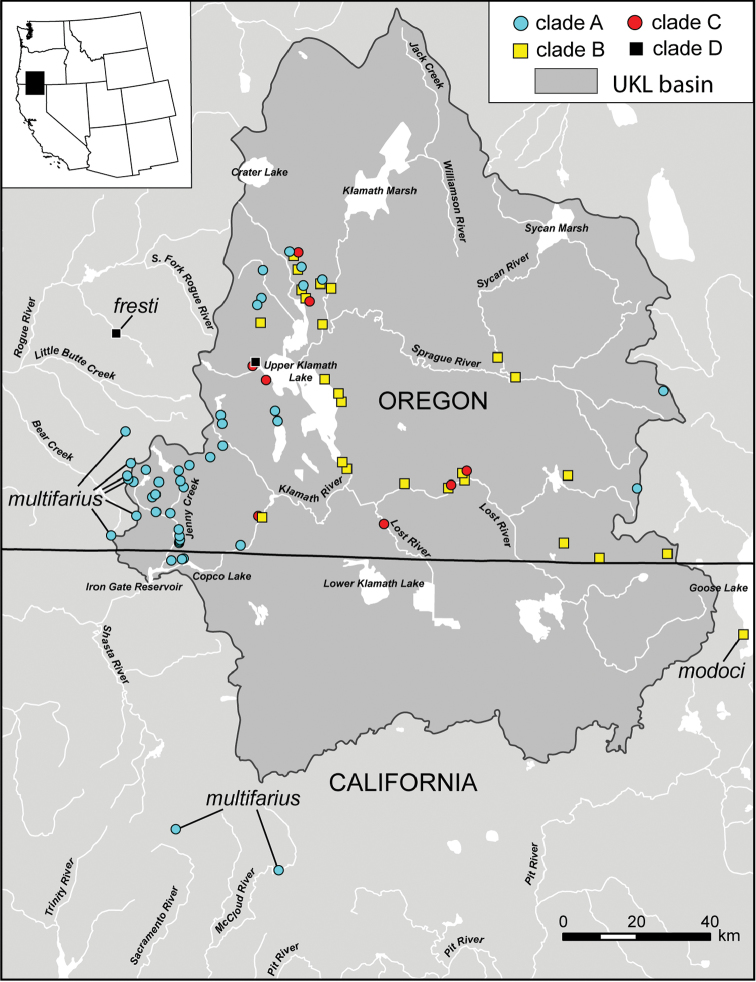
Map of southwest Oregon and northwest California showing the distribution of mtDNA clades **A–D** with color codes matching those in Figure 1.

Ninety-four (94) COI and 96 cytB haplotypes were detected in the analyzed UKL specimens (Suppl. materials 2, 3, respectively). The molecular phylogenetic and distance analyses of the two datasets generated closely similar trees in which the UKL haplotypes were resolved into four clades. Three of the clades were strongly supported (>95% bootstrap or posterior probability) in all analyses, while the fourth (clade A) was strongly supported only in the cytBBI analysis. The BI topology based on the COI sequences is shown in Figure 1 and the geographic distributions of the four clades are shown in Figure 2. Pairs of the lineages were sympatric at eight localities and three of the lineages co-occurred at one site (Wood River south headsprings).

Clade C is composed of pebblesnails from eight UKL localities. This lineage is well differentiated genetically from currently recognized *Fluminicola* species (7.6–17.2% for COI, 6.4–20.3% for cytB) and is further distinguished by unique penial morphology; we describe it as a new species below. The phylogenetic relationships of this new species were not well resolved.

**Figure 3.**
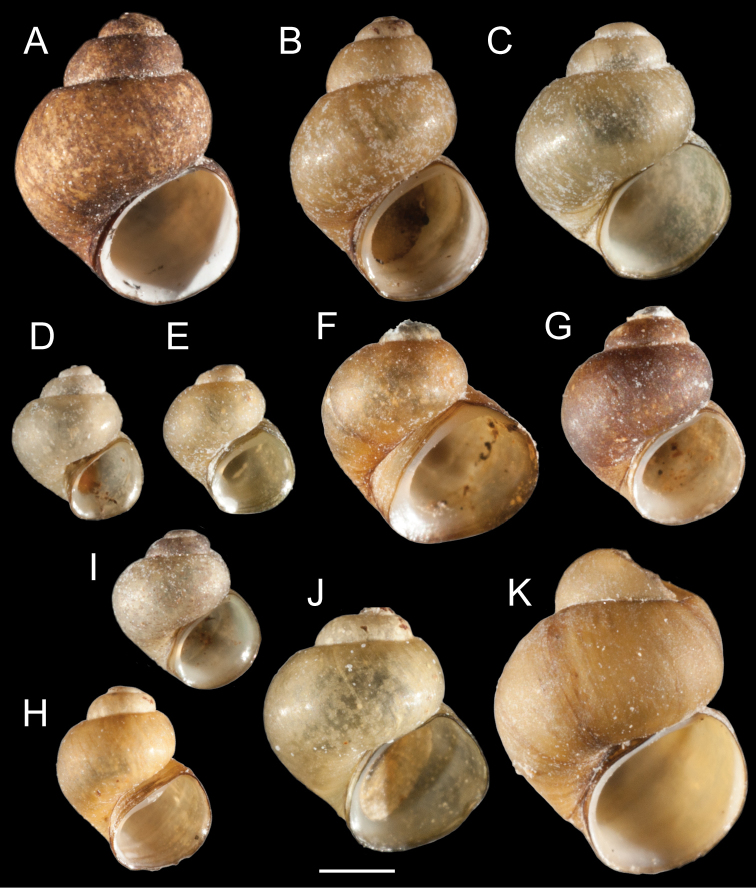
Shells of UKL*F.multifarius***A** USNM 1144951 **B** USNM 1144587 **C** USNM 1190091 **D, E** USNM 1020970 **F, G** USNM 1145066 **H** USNM 1144903 **I** USNM 1190128 **J** USNM 1190104 **K** USNM 1144463. Scale bar: 1.0 mm.

**Figure 4.**
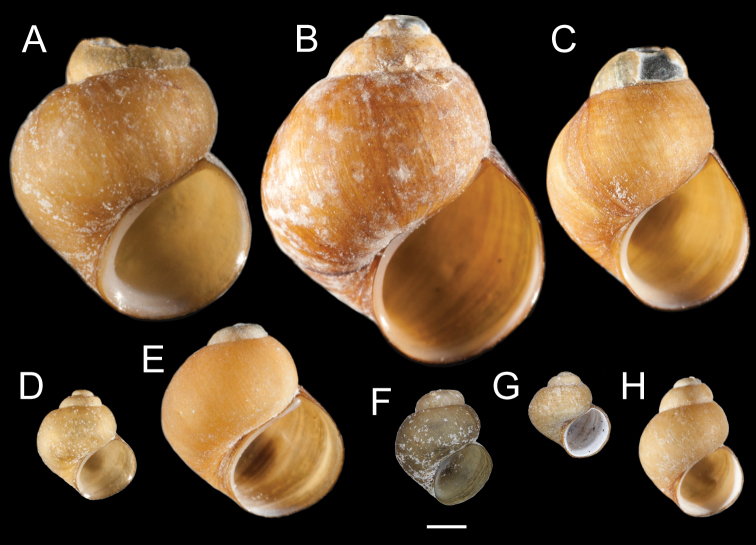
Shells of UKL*F.modoci* (**A–G**) and *F.fresti* (**H**) **A** USNM 1144454 **B** USNM 1144520 **C** USNM 1144942 **D** USNM 1144407 **E** USNM 1144966 **F** USNM 1190096 **G** USNM 1144565 **H** USNM 1144900. Scale bar: 1.0 mm.

Clades A, B and D contain both UKL pebblesnails and currently recognized species from other regional drainages (Figs 1, 2). Clade A contains *F.multifarius* and a large number of UKL populations varying in shell size and shape (Fig. 3). Although most of the UKL pebblesnails in this clade have subglobose to narrowly conical shells conforming to *F.multifarius*, populations in the Jenny Creek drainage often contain additional forms that fall outside of the range of variation previously reported for this species (e.g., Fig. 3F, shell neritiform; Fig. 3H, shell having a distinct swelling on the inner apertural lip). Clade B contains *F.modoci* and UKL pebblesnails that well conform to this species aside from having a much larger range in shell size among populations (Fig. 4A–G). Clade D is composed of *F.fresti* Hershler, Liu & Hubbart, 2017 and pebblesnails from Harriman Springs that closely resemble this species in their shells (Fig. 4H).

The sequence divergence between the UKL pebblesnails and the extra-limital species in clades A, B, and D is <4% for both genes (Table 1), which is relatively small compared to that among currently recognized *Fluminicola* species, which ranges from 1.7–18.7% for COI and 1.4–25.7% for cytB, with 93% (301/325) and 96% (288/300) of the pairwise comparisons >4%, respectively. The morphologically diverse UKL pebblesnails in both clades A and B are also relatively little differentiated genetically (clade A, mean sequence divergence 2.2% for COI and 2.9% for cytB; clade B, 0.4% for both genes). (Note that clade B was shallowly structured in all the trees while clade A was somewhat structured only in the BI topologies.) We also found that the pronounced phenotypic variation within populations (belonging to clade A) in the Jenny Creek drainage is not accompanied by substantial genetic divergence. For example, specimens having neritiform, simple conical, and conical shells with a swelling on the inner apertural lip that were collected in sympatry at the Fall Creek locality (UKL 50–52) differ by only 0.6–0.7% for COI, and 0%–0.5% for cytB. Based on this body of evidence we recognize clades A, B and D as single species corresponding to *F.multifarius*, *F.modoci*, and *F.fresti*.

**Table 1.** Sequence divergence within and between the extra-limital and UKL components of clades A, B, and D. Values are mean ± standard deviation.

	**COI**		**cytB**	
Clade A	UKL	* F. multifarius *	UKL	* F. multifarius *
UKL	2.2 ± 0.3%		2.9 ± 0.4	
* F. multifarius *	2.3 ± 0.3%	2.0 ± 0.4%	3.3 ± 0.5	3.1 ± 0.5
Clade B	UKL	*F.modoci* (D42)	UKL	*F.modoci* (D42)
UKL	0.4 ± 0.1%		0.4 ± 0.1%	
*F.modoci* (D42)	1.0 ± 0.3%	–	1.2 ± 0.5%	–
Clade D	UKL25A	* F. fresti *	UKL	* F. fresti *
UKL25A	–		–	
* F. fresti *	3.8 ± 0.7%	2.2 ± 0.3%	3.9 ± 0.6%	2.2 ± 0.3%

UKL25A consists of a single sequenced specimen.

## Taxonomic treatments

### Family Lithoglyphidae Troschel, 1857

#### Genus *Fluminicola* Carpenter, 1864

##### Fluminicola klamathensis

Taxon classificationAnimaliaAnnulatascalesAnnulatascaceae

Liu & Hershler sp. n.

http://zoobank.org/CC65E345-A320-4B5D-A267-EAF676F7B950

**Figure 5.**
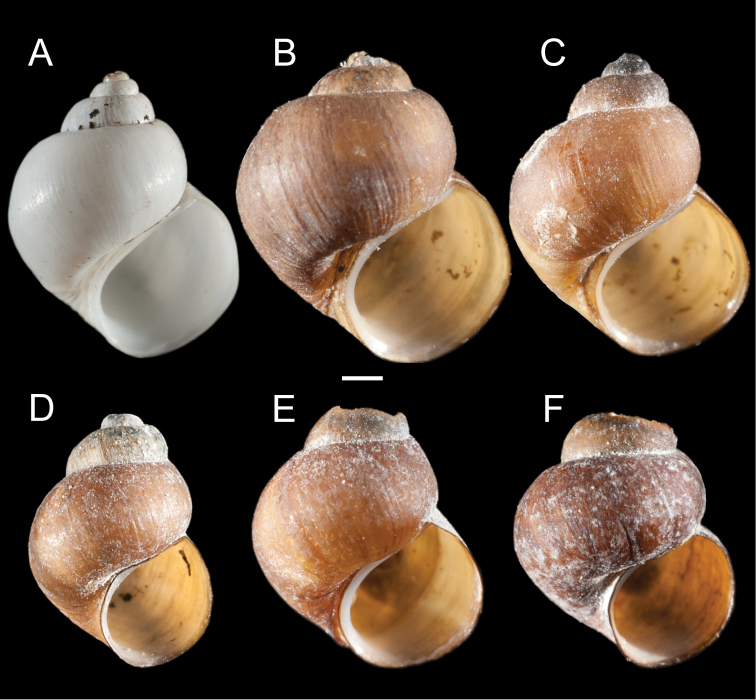
Shells of *F.klamathensis*, sp. n. **A** Holotype, USNM 144499 **B, C** USNM 1469076 **D** USNM 1469078 **E** USNM 1144346 **F** USNM 1144894. Scale bar: 1.0 mm.

**Figure 6.**
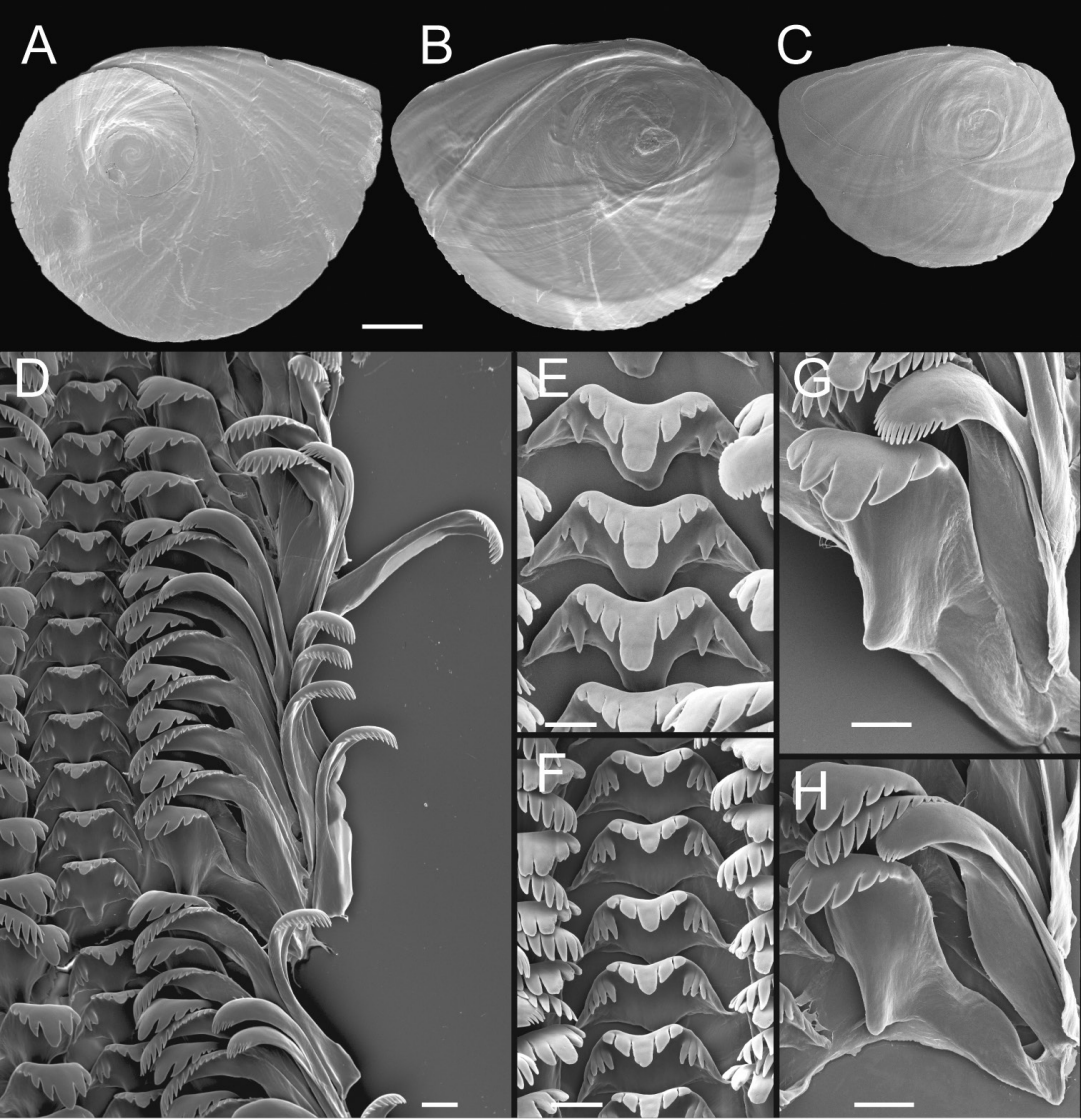
Opercula and radula, *F.klamathensis*, sp. n. **A, B** Opercula (outer, inner sides), USNM 1468970 **C** Operculum (inner side), USNM 144348 **D** Portion of radular ribbon, USNM 1468970 **E, F** Central teeth, USNM 144346, USNM 1144348 **G, H** Lateral teeth, USNM 1144346, USNM 1144348. Scale bars: 500 µm (**A–C**); 20 µm (**D–H**).

**Figure 7.**
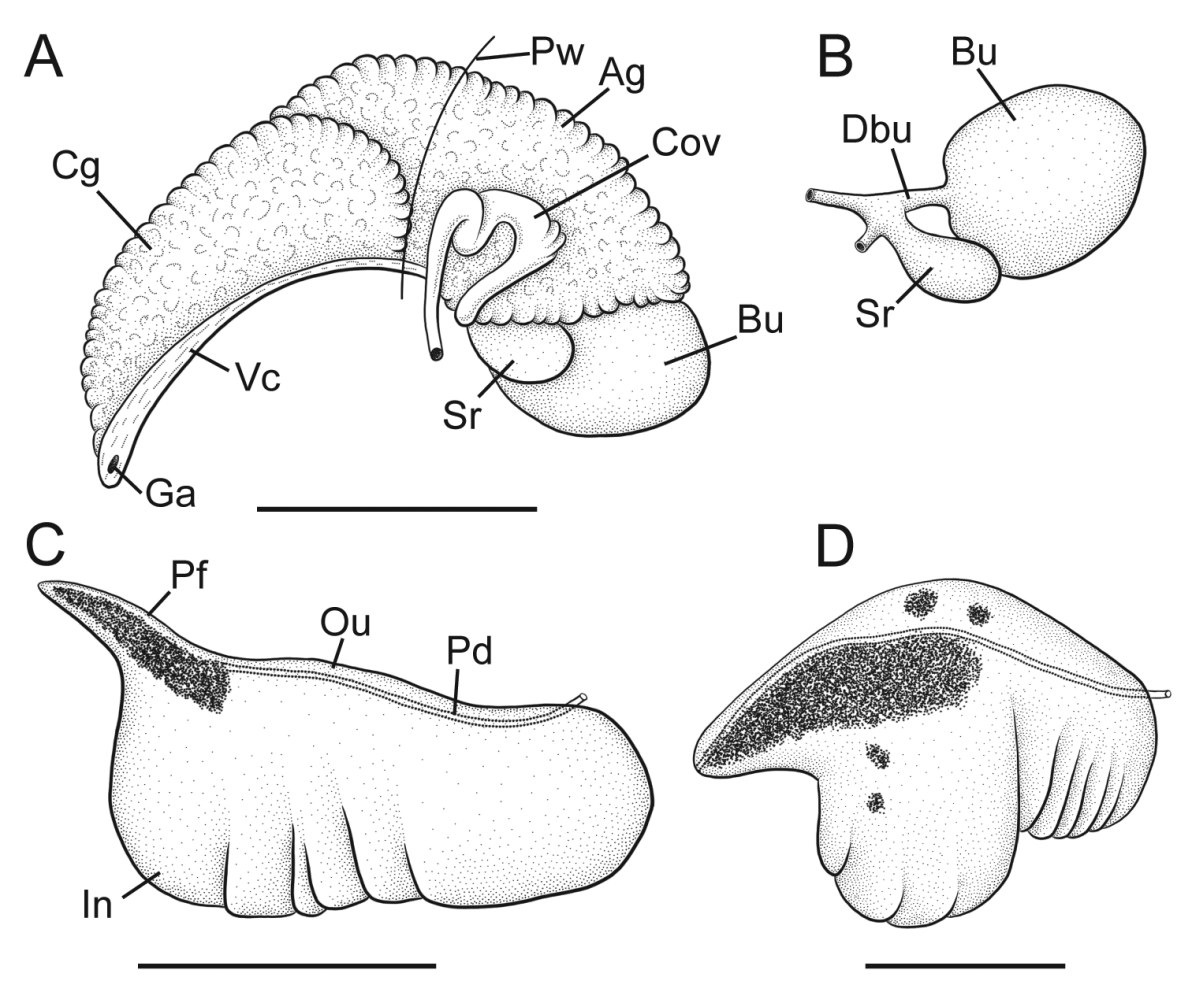
Reproductive anatomy, *F.klamathensis* sp. n. **A** Female glandular oviduct and associated structures (viewed from left side), USNM 1225874 **B** Bursa copulatrix and seminal receptacle, USNM 1225874 **C, D** Penis (dorsal surface), USNM 1225874, USNM 1469090. **Ag** albumen gland **Bu** bursa copulatrix **Cg** capsule gland **Cov** coiled oviduct **Dsr** seminal receptacle duct **Ga** genital aperture **In** inner edge of penis **Ou** outer edge of penis **Pd** penial duct **Pf** penial filament **Pw** posterior wall of pallial cavity **Sr** seminal receptacle **Vc** ventral channel of capsule gland. Scale bars: 1.0 mm (**A–C**); 500 µm (**D**).

**Figure 8.**
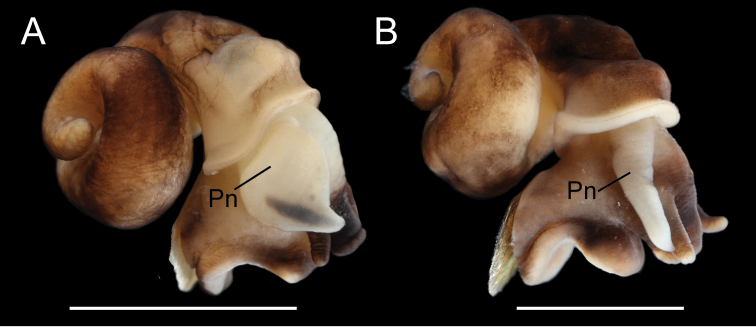
Photographs of ethanol preserved, relaxed specimens of *F.klamathensis* sp. n. (**A**) and *F.modoci* (**B**) from the Wood River south headspring, showing the differences in penial morphology **A** USNM 1469072 **B** USNM 1144736. **Pn** penis. Scale bar: 200 µm.

Figures 5 , 6 , 7 , 8A

###### Types.

Holotype, USNM 1144499 (a cleaned shell), Lost River at Stukel Bridge, Klamath County, Oregon, 42.0825N, 121.6617W, 10/5/1997, Terrence J. Frest and Edward J. Johannes. Paratypes, USNM 1468970 (a large series of dry shells and alcohol-preserved specimens), from same lot.

###### Referred material.

OREGON. *Klamath County*: USNM 1207966, Lost River at Stukel Bridge, USNM 1144894, USNM 1469075, USNM 1469082, USNM 1469090, Wood River, south spring source (42.7372N, 121.9775W), USNM 1469072, USNM 1469077, USNM 1469078, USNM 1469080, Tecumseh Spring (42.6424N, 121.9432W), USNM 1144346, USNM 1190088, Camporee Spring (42.4308N, 122.0614W), USNM 883517, USNM 1144348, USNM 1190089, USNM 1207965, USNM 1225874, Harriman Spring, outflow of main spring (42.4673N, 122.1009W), USNM 1469076, USNM 1469086, Big Springs at Bonanza (42.1982N, 121.4004W), USNM 1469074, USNM 1469088, Lost River, below Harpold Dam (42.1702N, 121.4530W).

###### Diagnosis.

A large *Fluminicola* (shell height, 6.5–8.4 mm) with a subglobose to ovate-conic shell often having an eroded spire. *Fluminicolaklamathensis* is readily distinguished by its penis, which does not gently taper along its length as in other congeners, but instead abruptly narrows distally and has a well demarcated, short filament. This new species is further differentiated from closely similar and frequently sympatric *F.modoci* in its generally darker colored shell periostracum, broad central cusps on the central and lateral radula teeth, very short outer wing of the lateral teeth, small number of cusps on the inner and outer marginal teeth, light pigment on the dorsal surface of the penis, large core of internal dark pigment in the distal section of the penis, and the fairly large seminal receptacle.

###### Description.

Shell (Fig. 5A–F) subglobose to ovate-conic, spire often eroded in large part, whorls (in specimens having a complete spire), 4.25–4.5. Teleoconch whorls low to medium convex, sometimes weakly shouldered. Aperture pyriform; inner lip complete, variably thickened, broadly adnate to parietal wall; columellar shelf narrow or extending over much of umbilical region. Outer lip thin, prosocline. Umbilicus absent or a narrow slit, umbilical region sometimes excavated. Shell white, periostracum brown, fairly thick, sometimes covered with black deposits. Shell measurements and whorl count data are summarized in Table 2.

**Table 2.** Shell parameters for *F.klamathensis*. Measurements are in mm.

	**WH**	**SH**	**SW**	**HBW**	**WBW**	**AH**	**AW**
Holotype, USNM 11444996							
	4.50	7.07	5.43	5.78	4.32	3.93	3.43
Paratypes, USNM 1468970 (*N* = 5)							
Mean	4.40	6.68	5.53	5.53	4.27	3.94	3.38
S.D.	0.14	0.52	0.48	0.36	0.30	0.38	0.25
Range	4.25–4.50	6.10–7.12	4.93–6.10	5.12–5.81	3.93–4.58	3.51–4.39	3.06–3.68

Operculum (Fig. 6A–C) as for genus; muscle attachment margin little thickened on inner side; rim sometimes present on inner side near outer edge (Fig. 6B). Radula (Fig. 6D–H) as for genus; dorsal edge of central teeth concave, lateral cusps 2–5, hoe-shaped; basal cusp 1–5. Lateral teeth having 2–4 cusps on inner side and 3–5 cusps on outer side; outer wing slightly longer than length of cutting edge. Inner marginal teeth with 11–19 cusps, outer marginal teeth with 11–22 cusps. Radula data are from USNM 144346, USNM 1468970.

Snout, cephalic tentacles grey or black, pigment light around eyespots; pallial roof, visceral coil usually light brown; foot variably pigmented dorsally, sole pale. Ctenidial filaments 33–36 (*N* = 5), broadly triangular. Glandular oviduct and associated structures shown in Figure 7A, B. Coiled oviduct circular, anterior arm kinked, posterior arm sometimes having small accessory pouches containing sperm. Bursa copulatrix large, ovate or globular, partly overlapped by albumen gland. Bursal duct narrow, much shorter than bursa. Seminal receptacle medium-sized, pouch-like, partly overlapped by albumen gland. Albumen gland having small pallial component. Capsule gland slightly shorter than albumen gland. Genital aperture a small, terminal pore. Penis (Figs 7C, D, 8A) large, base rectangular, often having a distinct, lobe-like swelling along inner edge distally (Fig. 7D); distal end of penis blunt, with short, narrow, filament. Distal section of penis having dense core of internal black pigment; dorsal surface pale or lightly pigmented. Penial duct near inner edge, narrow, nearly straight.

###### Etymology.

The species name is an adjectival geographic epithet referring to the distribution of this pebblesnail in the (upper) Klamath River basin.

###### Distribution.

Large, spring-influenced habitats in the UKL.

###### Remarks.

We selected the Lost River at Stukel Bridge as the type locality because the pebblesnails in this population do not have apically eroded shells. However, we did not have suitably relaxed material from this locality for anatomical study and thus used specimens from Harriman Springs for this purpose.

The shells of *F.klamathensis* and *F.modoci* can be difficult to distinguish although the former usually has darker, thicker periostracum, and tends to be larger when found in sympatry. As noted above, these two species are most readily differentiated by the shape and pigmentation of the penis (Fig. 8).

Sixteen (16) COI and 13 cytB haplotypes were detected in *F.klamathensis* (Suppl. materials 2, 3, respectively).

The “Tall pebblesnail” (also referred to as *Fluminicola* n. sp. 2) that was recognized by Frest and Johannes in their UKL contract reports (also see Frest and Johannes 1999) and subsequently included in the Northwest Forest Plan as a Survey and Manage species, corresponds to *F.klamathensis*.

##### Fluminicola fresti

Taxon classificationAnimaliaAnnulatascalesAnnulatascaceae

Hershler, Liu, Frest & Hubbart, 2017

Figure 4H

Fluminicola fresti –Hershler et al. 2017: 10–14, figs 4E–G, 5 (Diversion from Big Butte Springs through Butte Falls Hatchery, just south of Butte Falls–Fish Lake Road (Jackson County 321) and 0.16 km west of Butte Falls–Prospect Road (Jackson County 922), Jackson County, Oregon, 42.5389N, 122.5551W).

###### Distribution.

North Fork Umpqua River drainage and Rogue River basin north of Little Butte Creek, Oregon (Hershler et al. 2017).

###### Referred material.

OREGON. *Klamath County*. USNM 1144900, USNM 1469091, Harriman Spring, outflow of main spring (42.4673N, 122.1009W),

###### Remarks.

The Harriman Springs pebblesnails have small (shell height, about 4.0 mm), narrowly conical shells with convex whorls that well conform to *F.fresti*; they also closely resemble this species in details of radula morphology. Radula: central teeth with 3–4 lateral cusps, 1 basal cusp; lateral teeth with 3–4 cusps on outer side, 2 cusps on inner side; inner marginal teeth with 23–28 cusps; outer marginal teeth with 28–36 cusps(USNM 1144900).

We sequenced only a single specimen of *F.fresti* from Harriman Springs, which was collected during our first visit to this locality. Our subsequent collections from this site that were preserved in 90% ethanol for mtDNA analysis did not contain this species although a few specimens were found in one of the subsamples that had been prepared for anatomical study.

This new record extends the range of *F.fresti* about 26 km eastward from the Rogue River headwaters.

##### Fluminicola modoci

Taxon classificationAnimaliaAnnulatascalesAnnulatascaceae

Hannibal, 1912

Figures 4 , 8B

Fluminicola modoci –Hannibal 1912: 187, pl. 8: fig. 30 (in part; Fletchers Spring, south end, Goose Lake, California).

###### Distribution.

Several springs in the Goose Lake basin, California–Oregon (Hershler and Frest 1996; Hershler 1999; Hershler et al. 2007).

###### Referred material.

OREGON. *Klamath County*. USNM 1190095, USNM 1207964, Wood River, south spring source (42.7372N, 121.9775W), USNM 1144333, USNM 1190094, Tecumseh Spring (42.6424N, 121.9432W), USNM 874187, USNM 874935, USNM 1144336, USNM 1144337, USNM 1144942, USNM 1190086, Barkley Spring (42.3822N, 121.8111W), USNM 1190102, Klamath River, east of Boyle Power Station (42.0934N, 122.0964W), USNM 1190087, Ouxy Spring (42.3989N, 121.8235W), USNM 1144390, USNM 1144923, USNM 1190100, Brown Spring (42.4951N, 121.2956W), USNM 1190093, Spring, Klamath Fish Hatchery (42.6519N, 121.9479W), USNM 1190096, Reservation Spring (42.7023N, 121.9629W), USNM 1144407, USNM 1190098, Spring, Williamson River campground (42.6584N, 121.8499W), USNM 1190097, Spring Creek, headspring (42.6690N, 121.8860W), USNM 1144411, USNM 1190090, Crystal Spring (42.5736N, 122.0823W), USNM 1144966, USNM 1190121, Big Springs at Bonanza (42.1982N, 121.4004W), USNM 1144418, USNM 1185800, Casebeer Spring, ouflow at Gerber Dam Road (42.2056N, 121.0592W), USNM 1144454, USNM 1190123, Duncan Spring, north complex (42.0416N, 121.0689W), USNM 1190123, Lost River at Big Springs City Park, Bonanza (42.1976N, 121.4002W), USNM 114469, USNM 1190124, Gwinn Spring Creek at Gwinn Spring Creek Road (42.0063N, 120.9545W), USNM 1020714, USNM 1144673, USNM 1144925, USNM 1154376, Link River under US97/OR140 bridge (42.2185N, 121.7892W). USNM 1190137, Link River, above Link River dam (42.2341N, 121.8036W), USNM 1144494, USNM 1144986, USNM 1207967, Lost River, Stevenson County Park (42.1831N, 121.5994W), USNM 1190139, Lost River, below Harpold Dam (42.1702N, 121.4530W), USNM 1144503, USNM 1190101, Sprague River at Beatty Gap (42.4476N, 121.2377W), USNM 1144515, USNM 1190099, Williamson River, Klamath County park (42.5705N, 121.8791W), USNM 1144520, USNM 1190140, Upper Klamath Lake, south of Modoc Point (42.4373N, 121.8672W). *Lake County*: USNM 1144565, USNM 1185798, Spring southeast of Slash Spring, Yocum Valley (42.0174N, 120.7316W).

###### Remarks.

The UKL*F.modoci* range from 2.6–8.4 mm in shell height and include specimens with eroded spires that closely resemble the type material for this species (e.g., Fig. 4A) as well as individuals with fully intact, ovate-conic to trochoidal shells. Radula: central teeth with 2–6 lateral cusps, 1–4 basal cusps; lateral teeth with 3–7 cusps on outer side, 2–5 cusps on inner side; inner marginal teeth with 13–30 cusps; outer marginal teeth with 20–43 cusps (USNM 1144337, USNM 1144418, USNM 1144925).

The new records extend the range of *F.modoci* about 130 km westward from the northwestern portion of the Goose Lake basin.

Twenty-seven (27) COI and 31 cytB haplotypes were detected in the UKL specimens of *F.modoci* (Suppl. materials 2, 3, respectively).

The “Klamath pebblesnail” (also referred to as *Fluminicola* n. sp. 1) that was recognized by Frest and Johannes in their UKL contract reports (also see Frest and Johannes 1999) and subsequently included in the Northwest Forest Plan as a Survey and Manage species, may correspond to *F.modoci*.

##### Fluminicola multifarius

Taxon classificationAnimaliaAnnulatascalesAnnulatascaceae

Hershler, Liu, Frest & Johannes, 2007

Figures 3 , 9

Fluminicola multifarius –Hershler et al. 2007: 415, 417, 419, figs 7M, 24, 25 (Big Springs (source) at Big Springs City Park northwest of the city of Mount Shasta, south of Spring Hill, Siskiyou County, California ([UTM zone 10] 556400 E, 4575265 N, 1092 m).

###### Distribution.

Sacramento River headwater region, upper reaches of the McCloud and Rogue River drainages, California–Oregon (Hershler et al. 2007; Hershler et al. 2017).

###### Referred material.

CALIFORNIA. *Siskiyou County*: USNM 1207974, Spring on Close Butte (41.9884N, 122.3229W), USNM 1190109, Spring northwest of Copco Reservoir (41.9873N, 122.3275W), USNM 1145066, USNM 1190108, Fall Creek above Copco Road bridge (41.9834N, 122.3623W). OREGON. *Jackson County*: USNM 1144324, USNM 1144325, USNM 1144326, USNM 1144898, USNM 1145117, USNM 1190128, USNM 1243229, USNM 1254453, Fredenburg Spring (42.1669N, 122.3268W), USNM 1207971, Spring Creek north of Schoolhouse Meadow (42.0357N, 122.3397W), USNM 1144903, USNM 1190105, USNM 1207969, Spring brook below Schoolhouse Meadow (42.0288N, 122.3374W), USNM 1144342, USNM 1144943, USNM 1144365, USNM 1144366, USNM 1144484, USNM 1190114, Keene Creek, east of bridge on Mill Creek Road (42.1046N, 122.4136W), USNM 1144368, USNM 1144718, USNM 1144946, USNM 1190103, Rattlesnake Spring (42.0625N, 122.3389W), USNM 1190104, Shoat Spring, source (42.0466N, 122.3360W), USNM 1207968, Shoat Springs, outflow near source (42.0456N, 122.3367W), USNM 1207970, Spring channel above Schoolhouse Meadow, adjacent to cabin ruins (42.0327N, 122.3379W), USNM 1144536, USNM 1144537, USNM 1144941, USNM 1144942, USNM 1190118, Spring north of Hyatt Reservoir (42.2064N, 122.4498W), USNM 1144540, USNM 1144541, USNM 1144907, USNM 1144993, USNM 1190131, Nameless Spring, outflow (42.2183N, 122.3087W), USNM 1190129, Bluejay Spring (42.1810N, 122.3368W), USNM 1144587, USNM 1190115, Spring, Chinquapin Mountain (42.1409N, 122.4268W), USNM 1190116, Spring along Beaver Creek Road (42.1467N, 122.4165W), USNM 1190117, Spring, Craine Prairie (42.1754N, 122.4086W), USNM 1190111, Spring north of Soda Spring (42.1025N, 122.3684W), USNM 1190119, Spring, west side of Burnt Creek Road (42.1761N, 122.4911W), USNM 1144655, USNM 1145079, USNM 1190130, Jenny Creek Spring (42.2034N, 122.3443W). *Klamath County*: USNM 1469081, USNM 1469089, Wood River, south spring source (42.7372N, 121.9775W), USNM 1190138, Spring west of Klamath River (42.0257N, 122.1351W), USNM 1144392, USNM 1144951, USNM 1190127, Tiger Lily Spring (42.6156N, 122.0935W), USNM 1144393, USNM 1190092, Four Mile Spring (42.6331N, 122.0778W), USNM 1469079, Spring, Klamath Fish Hatchery (42.6519N, 121.9479W), USNM 1469083, Reservation Spring (42.7023N, 121.9629W), USNM 1469084, Spring Creek, headspring (42.6690N, 121.8860W), USNM 1144414, USNM 1144965, USNM 1190091, Short Creek, headspring (42.7000N, 122.0776W), USNM 1144416, USNM 1207975, Rainbow Springs (42.3239N, 122.2040W), USNM 1190134, Cold Creek, south of Lake of the Woods (42.3434N, 122.2083W), USNM 1144463, USNM 1190133, Spring along western edge of Buck Lake (42.2670N, 122.1995W), USNM 1190132, Johnson Creek (42.2401N, 122.2399W), USNM 1144468, USNM 1190136, Spring (northernmost), Denny Creek (42.3552N, 122.0286W), USNM 1144887, USNM 1144984, USNM 1190135, Spring (southernmost), Denny Creek (42.3324N, 122.0221W). *Lake County*: USNM 1190125, Spring, Holmes Meadow (42.1761N, 120.8350W), USNM 1144564, USNM 1185799, Blonde Spring (42.4149N, 120.7467W).

###### Remarks.

**Figure 9.**
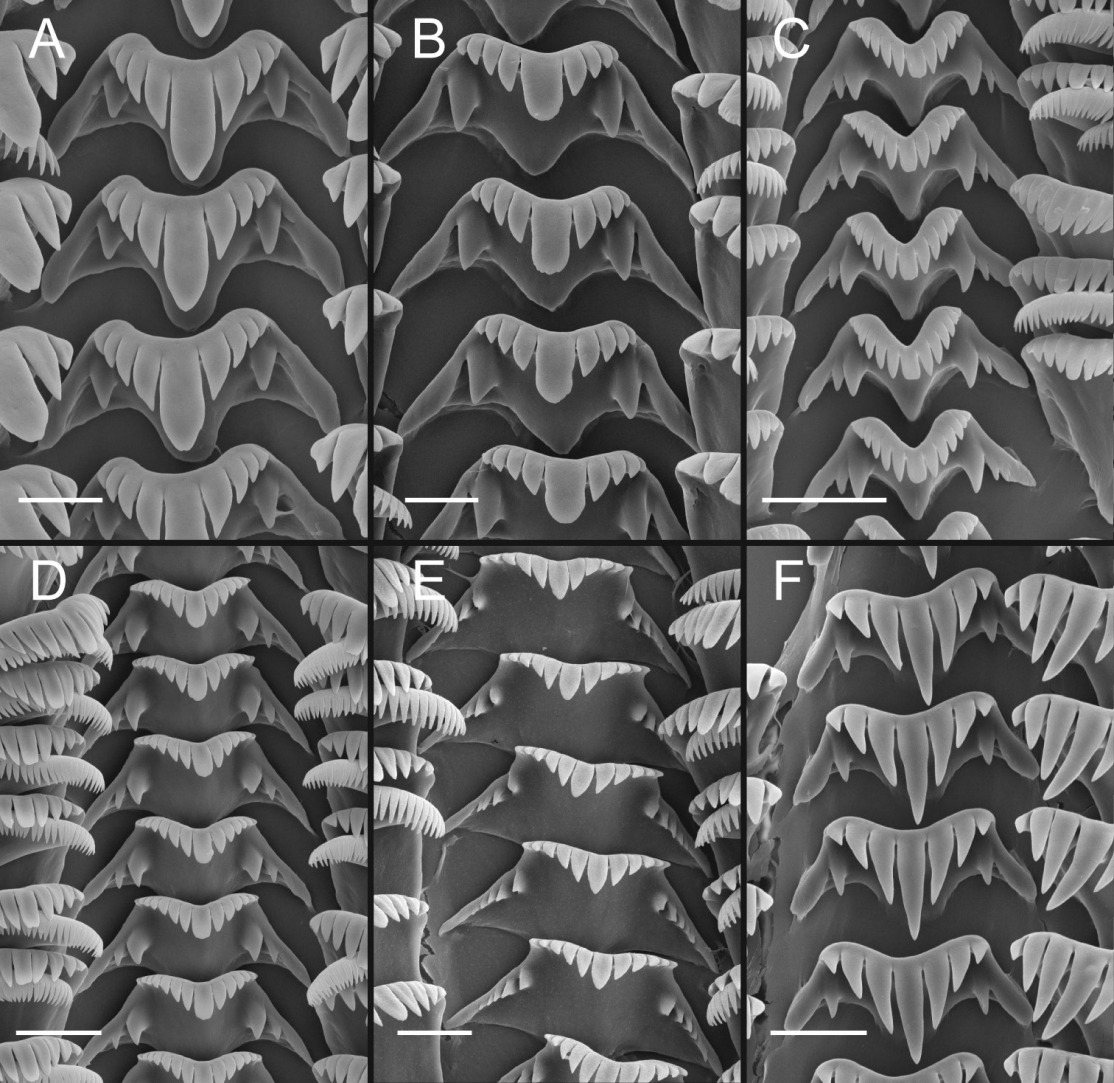
Variation in central radular teeth, UKL*F.multifarius***A, C, E** USNM 1207968 **B** USNM 144951 **D** USNM 1144368 **F** Central teeth, USNM 1207970. Scale bar: 10 µm.

The UKL*F.multifarius* range from 2.1–5.1 mm in shell height and, as noted above, vary considerably in shell shape and appearance of the inner apertural lip between and sometimes within populations. There is also considerable variation in the number of cusps on the radular teeth; and the size and shape of the cusps and indentation of the dorsal edge of the central teeth (Fig. 9). Radula: central teeth with 2–6 lateral cusps, 1–6 basal cusps; lateral teeth with 3–7 cusps on outer side; 2–6 cusps on inner side; inner marginal teeth with 16–35 cusps; outer marginal teeth with 22–40 cusps (USNM 1144326, USNM 1144342, USNM 1144368, USNM 1144463, USNM 1144540, USNM 1144564, USNM 1144588, USNM 1144951, USNM 1145079, USNM 1207968, USNM 1207970, USNM 1207974).

Fifty-one (51) COI and 52 cytB haplotypes were detected in the UKL specimens of *F.multifarius* (Suppl. materials 2, 3, respectively).

The “Fredenburg pebblesnail” (also referred to as *Fluminicla* n. sp. 17) and “Klamath Rim pebblesnail” (also referred to as *Fluminicola* n. sp. 3), which were recognized by Frest and Johannes in their UKL contract reports (also see Frest and Johannes 1999) and subsequently included in the Northwest Forest Plan as Survey and Manage species, correspond to *F.multifarius*.

The new records detailed herein extend the range of *F.multifarius* about 150 km eastward from the Rogue River headwaters. Populations of this species are distributed in close proximity (ca 1 km) across the divide between the Rogue River and UKL basins (springs in Sampson Creek and Burnt Creek drainages, respectively).

## Discussion

Our findings, based on both morphologic and genetic (mtDNA sequences) evidence, have shown that contrary to previous assertions in the grey literature (Frest and Johannes 1998, 2000, 2004, 2005), the UKL does not have a large, highly endemic fauna of undescribed pebblesnails, but instead contains only four species, one of which is new and three of which were previously described from other regional drainages. Although we only surveyed 58 of the >200 UKL pebblesnail localities reported by Frest and Johannes, we sampled at least one locality for each of the putatively new species that they recognized, and we sampled numerous localities in the Jenny Creek watershed where much of the phenotypic diversity of UKL pebblesnails is concentrated. Thus, we are confident that we have well delineated the taxonomic diversity of UKL pebblesnails. Additional studies will be needed to further delineate the distributions of the four species in the UKL and to determine whether these pebblesnails range into the lower reach of the large Klamath River watershed.

Our study also provides evidence of striking morphologic variation in *F.modoci* and *F.multifarius* similar to what has been observed in various marine caenogastropod lineages (e.g., *Littorina*; Reid 1996). Further investigations utilizing rapidly evolving nuclear markers such as microsatellites should provide additional insight into the apparent decoupling of morphologic and genetic variation in these two species and, together with ecological studies, help tease apart the underlying mechanisms for the sympatric occurrence of multiple *F.multifarius* morphotypes at various localities in the Jenny Creek area.

## Supplementary Material

XML Treatment for Fluminicola klamathensis

XML Treatment for Fluminicola fresti

XML Treatment for Fluminicola modoci

XML Treatment for Fluminicola multifarius

## Supplementary materials

Supplementary material 1 Table S1 Data type: specimen data Explanation note: Sample codes, USNM voucher numbers (for newly sequenced specimens), locality details, and GenBank accession numbers for COI and cytB sequences. This is an open access article distributed under the terms of the Creative Commons Attribution License (CC BY 4.0), which permits unrestricted use, distribution, and reproduction in any medium, provided the original author and source are credited.Hsiu-Ping Liu, Robert Hershler

Supplementary material 2 Table 2 Data type: molecular data Explanation note: Distribution of COI haplotypes. Exemplars used in the phylogenetic analyses are in parentheses. This is an open access article distributed under the terms of the Creative Commons Attribution License (CC BY 4.0), which permits unrestricted use, distribution, and reproduction in any medium, provided the original author and source are credited.Hsiu-Ping Liu, Robert Hershler

Supplementary material 3 Table S3 Data type: molecular data Explanation note: Distribution of cytB haplotypes. Exemplars used in the phylogenetic analyses are in parentheses. This is an open access article distributed under the terms of the Creative Commons Attribution License (CC BY 4.0), which permits unrestricted use, distribution, and reproduction in any medium, provided the original author and source are credited.Hsiu-Ping Liu, Robert Hershler
